# Activating p53 in Cancer Cells with Protein Therapy Shows Preclinical Promise

**DOI:** 10.1371/journal.pbio.0020058

**Published:** 2004-02-17

Late-stage cancers are notoriously unresponsive to treatment, making certain hard-to-detect cancers particularly insidious. Ovarian cancer, for example, most often escapes diagnosis until the tumor has already metastasized. At this stage, ovarian cancer is classified as peritoneal carcinomatosis, a terminal condition characterized by widespread tumor growth throughout the peritoneum, the large serous membrane that lines the abdominal cavity, pelvis, and associated organs. Advanced cases of peritoneal carcinomatosis are largely resistant to chemotherapy and account for the bleak 15%–20% survival rate of ovarian cancer.

Biologists often view cancer as an evolutionary process in which cells that would normally cooperate with their neighbors begin to compete with them. Selective advantage for cancer cells often begins with mutations that inhibit tumor suppressor pathways. p53, like other tumor suppressor genes, arrests cell growth and induces apoptosis (programmed cell death) in response to cellular stress, such as chromosomal damage. Cells with p53 mutations often escape these constraints, leading to the uncontrolled growth characteristic of “immortal” cancer cells. Nearly all types of tumors have mutations in the p53 pathway. Treatments focused on restoring p53 function—which is likely to be defective only in cancer cells—should prove more effective than chemotherapies, which indiscriminately kill all dividing cells, healthy or cancerous. With the goal of developing targeted therapeutic strategies, Steven Dowdy and colleagues show that restoring p53 protein function in tumor cells not only dramatically increases lifespan in mice but also eliminates disease.

While past efforts to restore tumor suppressor function in cancer cells have focused on gene therapy, Dowdy and colleagues introduced modified p53 peptides, or protein fragments, into cancer cells. p53 works as a “transcriptional” activator that binds to specific sequences of DNA and triggers apoptosis in response to DNA damage. Its biological function flows from this binding ability. One region of this large protein, called the C-terminal domain, facilitates effective binding. In cancer cells, synthesized peptides (called p53C′) derived from this region can induce apoptosis by activating p53—which is normally present in low levels in a biologically inactive form—and restoring function to p53 proteins with DNA-binding mutations.

To get p53C′ peptides into cancer cells, Eric Snyder et al. used a technique pioneered by Dowdy that delivers large proteins into the cell interior. Since the cell membrane normally limits passage to only small molecules (larger molecules generally enter through surface receptors), this is no small feat. The technique exploits the ability of a small peptide region from the HIV TAT protein to smuggle macromolecules through cell membranes that normally prohibit entry to such large molecules. After synthesizing a structurally modified form of p53C′ less prone to degradation, the researchers first confirmed that the peptide was functional and then that it activates p53-specific genes in tumor cells, but not in normal cells. Testing the effectiveness of the peptide therapy on mouse strains used to model human metastatic disease, they found that mice treated with the TATp53C′ peptide showed a significant reduction in tumor growth and lived six times longer than both mice treated with a control peptide and untreated mice, with some mice remaining disease-free more than 200 days after treatment.

This macromolecular delivery approach, Snyder et al. argue, works with greater specificity and avoids the tumor-generated neutralizing effects observed in small molecule strategies. Because a mutation in the p53 gene is one of the most common events in the development of cancer, these results could have implications for a wide variety of cancers. And by working with mouse models that approximate the physiological burdens metastatic cancer imposes on humans, Dowdy's team demonstrates the promise of developing targeted “intracellular biologic” therapeutics that treat the systemic pathology of cancer—inhibiting tumor growth as well as alleviating the lethal complications associated with the disease.

**Figure:**
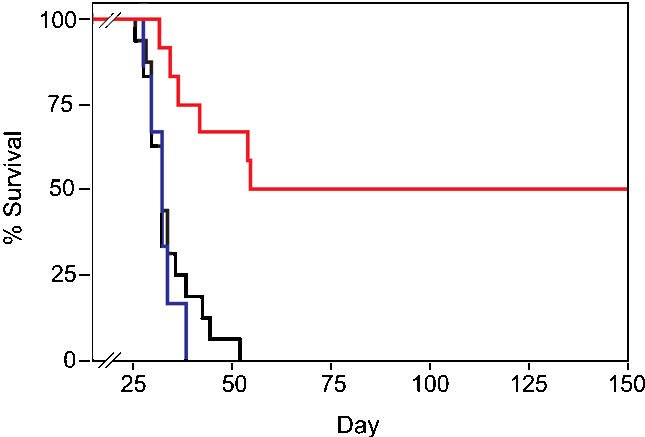
TATp53C′ treatment extends survival of mice

